# Puerperal Convulsions

**Published:** 1874-07

**Authors:** J. J. Elan


Puerperal Convulsions.—*By J. J. Elan, M.D.*—* * * Dr. Wallace fully agreeing with me, a *coal of fire* was procured—the patient being at the time in a severe convulsion—and passed rapidly over the chest, making a series of small blisters, the largest over the region of the heart. Reaction came on immediately, and the jaws, previously clenched, now moved freely, and the patient exclaimed, "Quit burning me!" * * *

The points worthy of special consideration in the foregoing case are:

1. The influence exerted over the circulatory and muscular systems by fire, as manifested by the increase in the volume and reduction in the frequency of the pulse, and the relaxation of the jaws previously locked.

2. The power of chloroform to cut short but not prevent the recurrence of the convulsions.

3. The action of chloral hydrate as a hypnotic. The action of fire I consider of the first importance, from the fact that chloroform, chloral, etc., though carefully and persistently employed, had failed.

